# New parathyroid function index for the differentiation of primary and secondary hyperparathyroidism: a case-control study

**DOI:** 10.1186/s12902-019-0487-8

**Published:** 2020-01-08

**Authors:** Yanhong Guo, Qin Wang, Chunyan Lu, Pianpian Fan, Jing Li, Ximing Luo, Decai Chen

**Affiliations:** 1 Endocrinology Department of West China Hospital, Sichuan University, Chengdu, China; 2 Endocrinology Department, Hospital of Chengdu Office of People’s Government of Tibetan autonomous Region, Chengdu, China

**Keywords:** Hyperparathyroidism, Parathyroid function index, Vitamin D deficiency

## Abstract

**Background:**

Patients with primary hyperparathyroidism (PHPT) may be asymptomatic, and some may present with normocalcemic PHPT (NPHPT). Patients with vitamin D deficiency may also be asymptomatic, with normal calcium and elevated PTH concentrations. These latter patients are usually diagnosed with vitamin D deficiency-induced secondary hyperparathyroidism (VD-SHPT). Therefore, it is very difficult to distinguish PHPT and NPHPT from VD-SHPT based on calcium or PTH concentrations in clinical settings. In this case-control study, we aimed to verify the diagnostic power of a new parathyroid function index (PFindex = Ca*PTH/P).

**Methods:**

This study enrolled 128 patients with surgically and pathologically confirmed PHPT, including 36 with NPHPT, at a hospital in West China between January 2009 and September 2017. Thirty-seven patients with VD-SHPT and 45 healthy controls were selected from the population of a cross-sectional epidemiological study as the SHPT and healthy groups, respectively. We used the PFindex to describe the characteristics of PHPT, NPHPT, and VD-SHPT.. Differences between the four groups were compared, and a receiver operating characteristic (ROC) curve analysis was used to evaluate the diagnostic power of PFindex.

**Results:**

The PHPT group had the highest PFindex (454 ± 430), compared to the other three groups (NPHPT: 101 ± 111; SHPT: 21.7 ± 6.38; healthy: 12.2 ± 2.98, all *p* < 0.001). A PFindex cut-off value of 34 yielded sensitivity and specificity rates of 96.9 and 97.6% and of 94.4 and 94.6% for the diagnoses of PHPT and NPHPT, respectively. The use of a PFindex > 34 to differentiate NPHPT from VD-SHPT yielded the highest positive likelihood ratio and lowest negative likelihood ratio.

**Conclusion:**

The PFindex provided excellent diagnostic power for the differentiation of NPHPT from VD-SHPT. This simple tool may be useful for guiding timely decision-making processes regarding the initiation of vitamin D treatment or surgery for PHPT.

## Background

Parathyroid hormone (PTH) is one of the most important hormones required for the maintenance of calcium and phosphate homeostasis. This hormone induces the 1α-hydroxylation of 25(OH) D to 1,25(OH)_2_D, which promotes intestinal absorption and the release of calcium and phosphate from the bone [1–3], while regulating mineral reabsorption in the renal tubules. The intrinsic abnormal excretion or extrinsic abnormal stimulation of PTH production leads to primary, secondary, or tertiary hyperparathyroidism [4].

A diagnosis of classic primary hyperparathyroidism (PHPT) can be made easily according to its biochemical, skeletal, and renal manifestations. However, increases in routine serum calcium testing, as well as the incidental discovery of parathyroid nodules on thyroid ultrasonography, has led to an increase in the detection frequency of asymptomatic PHPT (including normocalcemic PHPT, NPHPT) in recent decades. Thus, a correct clinical diagnosis of this disease is important.

Chronic renal insufficiency and vitamin D deficiency are the most common causes of secondary hyperparathyroidism (SHPT). The former can be easily distinguished from the medical history and laboratory tests. However, it is difficult to distinguish vitamin D deficiency-induced SHPT (VD-SHPT) from PHPT. Patients diagnosed with either VD-SHPT or PHPT would have an elevated PTH concentration [5], and many presented with vitamin D deficiency as well as a normal serum calcium concentration [6–8]. Therefore, it is difficult to distinguish between these diseases based on laboratory results. Although the two diseases can be distinguished when a patient’s vitamin D storage is replete, a period of at least 8 weeks is required to normalize the 25(OH) D concentration [9–11], leading to delays in appropriate treatment. Therefore, a convenient clinical tool to differentiate PHPT from VD-SHPT.

According to the pathogeneses of these two diseases, PHPT is associated with a relatively higher serum calcium but lower phosphate concentration, while VD-SHPT is characterized by relatively lower serum calcium and phosphate concentrations. Using this information, we created a parathyroid function index (PFindex) to magnify the biochemical differences between these diseases. This equation multiplies the serum PTH (pmol/L) by the albumin-corrected serum calcium concentration (mmol/L), and then divides this value by the serum phosphate concentration (mmol/L). In this case-control study, we aimed to verify the diagnostic power of the PFindex in subjects with confirmed PHPT and SHPT, as well as healthy subjects.

## Methods

### Study design

This retrospective case-control study included 92 patients with PHPT and elevated calcium levels, 36 with NPHPT, 37 with SHPT, and 45 healthy patients. Data were retrieved from the PHPT registry of the Department of Endocrinology at a single hospital in West China. Biochemical parameters were obtained from electronic medical records and compared among different groups. Control groups were selected from a community-based, cross-sectional study conducted in Sichuan Province, China. This study was approved by the Medical Ethics Committee of Sichuan University, which agreed to waive the requirement for informed consent.

### Study population

This study included 128 cases of pathologically confirmed PHPT, including 36 cases NPHPT, at a hospital in West China between January 2009 to September 2016. Overall, 108, 11, 7, and 2 patients had parathyroid adenoma, parathyroid hyperplasia, parathyroid carcinoma, and ectopic parathyroid, respectively. Thirty-seven age-matched cases of VD-SHPT and 45 age-matched controls were selected from a community-based, cross-sectional study conducted in Sichuan Province, China. Subjects with a serum PTH concentration > 6.9 pmol/L and 25-(OH) D concentration < 20 ng/mL were classified into the SHPT group. All healthy control subjects had a normal serum PTH concentration (normal laboratory range: 14.5–62.7 pg/mL). In addition, subjects in the two control groups met all the following inclusion criteria: T-scores less than − 1 at the femoral neck, total hip, or lumbar spine measured by dual energy x-ray absorptiometry (DXA); no history of fracture, kidney disease, severe scoliosis, or hyperosteogeny of the lumbar vertebra; and serum calcium and phosphorus concentrations within normal ranges.

### Assessment of PTH, 25-(OH) D, serum calcium, and phosphorous

The PTH and 25-(OH) D concentrations in the control groups were measured by our Laboratory Department using enzyme-linked immunosorbent assays (ELISAs; Immunodiagnostic Systems, IDS Ltd., London, UK). The interassay coefficients of variation (CV) were 4.7 and 4.6%, respectively. The serum calcium, phosphorous, and albumin concentrations were measured using colorimetric methods (CVs: 1.8, 1.5 and 1.5%, respectively). Corrected calcium was calculated as the measured calcium + (40 − measured albumin) * 0.02 [12]. The PFindex was calculated as follows: PFindex = Ca*PTH/P.

### Statistical analysis

SPSS statistical software was used for the data analysis (version 18.0.2; SPSS Inc., Chicago, IL, USA). Normally distributed continuous variables are presented as means ± standard deviations (SDs). Differences between groups were tested using one-way ANOVAs after normal transformation, and non-normally distributed data were evaluated with statistical disposal. A receiver operating characteristic (ROC) curve analysis was performed to evaluate the diagnostic ability of the PFindex, and ROC curves were plotted to examine the balance between sensitivity and specificity. To compare the diagnostic value of the PFindex with the PTH and serum calcium concentrations, the Youden index was calculated (Youden index = sensitivity + specificity - 1). Statistical significance was defined as a *P*-value < 0.05.

## Results

### Characteristics of the subjects

**Fig. 1:**
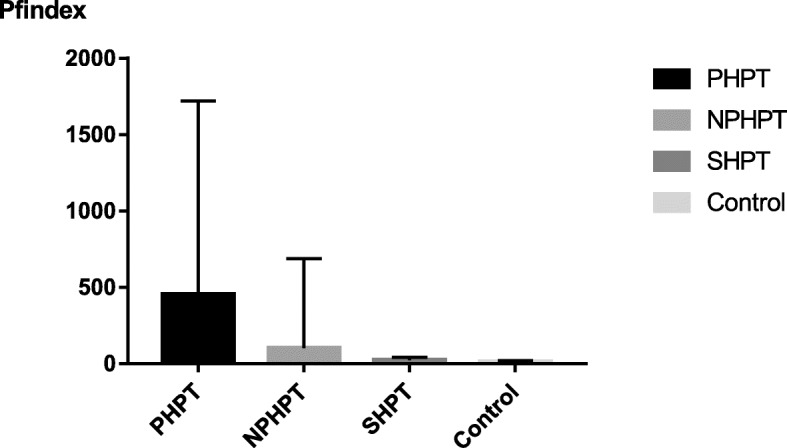
Pfindex value in 4 groups

The PHPT group had the highest corrected serum calcium, PTH, and PFindex values among the four groups (all *P* < 0.05). The 25(OH) D level was higher in the healthy group than in the PHPT, NPHPT, and SHPT groups (Table 1, Fig. 1).

**Table 1:** Characteristic of subjects in PHPT, NPHPT, SHPT and Health groups

	PHPT(*n* = 92)	NPHPT(*n* = 36)	SHPT(*n* = 37)	Healthy(*n* = 45)
Age (years)	47.7 ± 15.5 ^**b**^	53.7 ± 15.9	46.0 ± 12.1	45.1 ± 12.3
Gender(M/F)	36/56	10/26	37/0	45/0
Serum calcium (2.1–2.7 mmol/L)*	3.24 ± 0.82^**a**^	2.59 ± 0.10 ^**a**^	2.19 ± 0.07	2.21 ± 0.11
Serum phosphate (0.81–1.45 mmol/L)	0.77 ± 0.34^**a**^	0.79 ± 0.15 ^**a**^	1.06 ± 0.15	1.07 ± 0.15
PTH (14.5–62.7 pg/mL)	884 ± 863^**a**^	252 ± 239	94.8 ± 28.6	52.6 ± 9.09
25(OH)D (19.08–57.6 ng/mL)	11.7 ± 4.0^**a**^	14.0 ± 5.02^**a**^	13.8 ± 2.63^**a**^	17.6 ± 6.74
Calcium×PTH	395 ± 402	72.4 ± 69.0	23.3 ± 48.4	12.3 ± 48.4
Lg Calcium×PTH**	2.31 ± 0.46 ^**a**^	1.75 ± 0.26 ^**a**^	1.37 ± 0.01	1.12 ± 0.10
PFindex	454 ± 430	101 ± 111	21.7 ± 6.38	12.2 ± 2.98
LgPFindex**	2.44 ± 0.46 ^**a**^	1.87 ± 0.31 ^**a**^	1.35 ± 0.12	1.10 ± 0.11
Ca/P	4.63 ± 1.40 ^**a**^	3.35 ± 0.71 ^**a**^	2.11 ± 0.34	2.10 ± 0.35

*: serum calcium was corrected by albumin. **: normal transformation

^a^: *P*<0.01 ^b^: *P*<0.05

### ROC curves for the corrected serum calcium, phosphorus, PTH, calcium×PTH, and PFindex values

The plotted ROC curve for the PFindex yielded sensitivity and specificity rates of 96.9 and 97.6%, respectively, at values > 34. The PFindex curve yielded a higher Youden index than the serum calcium, phosphorus, PTH, and Calcium×PTH curves (Tables 2, 3).

**Table 2:** Sensitivity, Specificity and Youden index of serum calcium, PTH, Calcium×PTH and Pfindex in diagnosis of PHPT

	Sensitivity (%)	Specificity (%)	Youden index
PFindex> 34	96.9	97.6	0.945
Calcium> 2.51	95.3	97.6	0.929
PTH > 11.96	85.1	100	0.851
Calcium*PTH > 35.43	93.0	97.6	0.906
Ca/*P* > 2.71	91.4	93.9	0.853

**Table 3:** Sensitivity, Specificity and Youden index of serum calcium, PTH, Calcium×PTH and Pfindex in diagnosis of NPHPT

	Sensitivity (%)	Specificity (%)	Youden index
PFindex> 34	94.4	94.6	0.890
Calcium> 2.45	91.7	89.2	0.809
PTH > 11.71	94.4	75.7	0.701
Calcium*PTH > 31.73	91.7	86.5	0.782
Ca/P > 2.71	82.2	91.9	0.741

## Discussion

The PFindex was the first index designed to quantify the parathyroid function and differentiate PHPT from SHPT. Based on our findings, the Youden index and positive likelihood ratio of the PFindex were higher than those of the Wisconsin Index. Consequently, this comprehensive index is superior to either single parameters or pairs thereof, due to its ability to reflect the interactions of serum calcium and phosphorus concentrations with PTH.

In a previous study, HaggiMazeh et al. designed the Wisconsin Index [13] by multiplying the preoperative serum calcium by the PTH concentration [13]. However, this index was not used for differential diagnosis, but rather was used to help surgeons determine whether to explore the neck further or wait for PTH results after minimally invasive parathyroidectomy. In another study by Madeo et al., a Ca/P ratio of 2.71 was considered valuable for the diagnosis of PHPT [14], consistent with our study. However, in our study, the diagnostic power of the PFindex was stronger than those of the Ca/P Ratio and Wisconsin Index (Tables 2, 3). The Wisconsin Index yielded a lower Youden index value than the PFindex for the diagnosis of NPHPT and PHPT, partly because it is incorrect to assess an imbalance in calcium and phosphate homeostasis only according to the PTH level, especially when using non-optimal reference intervals for serum PTH. The Youden index of the Ca/P Ratio was lower than those of both the PFindex and Wisconsin Index for the diagnosis of NPHPT and PHPT. Although the diagnosis of PHPT is based generally on an elevated PTH level combined with an elevated or normal calcium level, the Ca/P Ratio ignores the PTH level.

The symptoms of PHPT are mainly attributable to an elevated calcium concentration. However, many PHPT patients are asymptomatic [15–17]. This phenomenon is partly explained by a decrease in serum calcium concentrations due to vitamin D deficiency. In previous studies, a high prevalence of vitamin D deficiency was observed in both healthy individuals [18, 19] and PHPT patients [6, 7]. Thus, the third International Workshop guidelines recommended the exclusion of vitamin D deficiency-induced SHPT prior to the diagnosis of PHPT [20]. It would be better to make a final diagnosis after correcting the vitamin D deficiency. As noted previously, vitamin D repletion requires a period of 2–3 months [9]. A PFindex > 34 could avoid this additional waiting time and allow the arrangement of advanced tests for PHPT, such as parathyroid ultrasound and radionuclide imaging. Patients with suspected hyperparathyroidism and a PFindex < 34 could take vitamin D supplements first instead of having expensive parathyroid tests such as SPECT. Thus, the simple calculation required to generate the PFindex might conserve medical resources during the diagnosis of hyperparathyroidism.

This study was subject to several limitations. First, there is no gold standard for the inclusion of SHPT subjects in our study. Patients with SHPT and healthy controls were selected from an epidemiology study of 1500 female residents of southwestern China, which has a low level of daily solar exposure [21]. Notably, the prevalences of vitamin D deficiency and vitamin D deficiency plus elevated PTH (PTH>6.9 pmol/L) in this population were 72.5 and 40.2%, respectively (unpublished data). Conversely, the prevalence of PHPT was so low (approximately 1–4 per 1000) that it was difficult to select PHPT patients [22]. Therefore, the recruited subjects with vitamin D deficiency and slightly elevated PTH concentrations were likely to be true SHPT patients. Second, the PHPT and control groups were not balanced with respect to sex, as only women were enrolled in the cross-sectional study. However, no evidence suggests that the reference ranges for serum calcium, phosphorus, and PTH differ between males and females. Finally, this study did not include children. Further studies are needed to determine whether the PFindex can be used to differentiate NPHPT from VD-SHPT in children.

## Conclusion

The PFindex, which assesses instability in calcium and phosphorus metabolism, was a more useful clinical diagnostic tool than serum calcium, phosphorus, or PTH alone. Specifically, Subjects with PHPT had a significantly higher PFindex than those with VD- SHPT, and the PFindex yielded a higher Youden index when compared with other indicators. A PFindex > 34 was identified as an appropriate differentiator between NPHPT and VD-SHPT, and this value yielded the highest positive and lowest negative likelihood ratios. This tool could help clinicians to differentiate PHPT from VD-SHPT.

## Data Availability

The dataset used in this study is available and can be provided upon written request (Yanhong Guo, Email: guoyanhong198911@163.com).

## Abbreviations

NPHPT: Normocalcemic PHPT
PFindex: Parathyroid function index
PHPT: Primary hyperparathyroidism
PTH: Parathyroid hormone
VD-SHPT: vitamin D deficiency induced secondary hyperparathyroidism
